# Sarcopenia as an independent predictor of the surgical outcomes of patients with inflammatory bowel disease: a meta-analysis

**DOI:** 10.1007/s00595-019-01893-8

**Published:** 2019-10-15

**Authors:** Adrienn Erős, Alexandra Soós, Péter Hegyi, Zsolt Szakács, Márton Benke, Ákos Szűcs, Petra Hartmann, Bálint Erőss, Patricia Sarlós

**Affiliations:** 1 Institute for Translational Medicine, Medical School, University of Pécs, 12 Szigeti Street, Pécs, 7624 Hungary; 2 Szentágothai Research Centre, University of Pécs, 20 Ifjúság Street, Pécs, 7624 Hungary; 3 Clinical Medicine Doctoral School, University of Szeged, 6 Korányi fasor, Szeged, 6720 Hungary; 4 Hungarian Academy of Sciences, University of Szeged Momentum Gastroenterology Multidisciplinary Research Group, 8-10 Korányi fasor, Szeged, 6720 Hungary; 5 First Department of Medicine, Medical School, University of Pécs, 13 Ifjúság Street, Pécs, 7624 Hungary; 6 First Department of Surgery, Semmelweis University, 78 Üllői Street, Budapest, 1082 Hungary; 7 Institute of Surgical Research, University of Szeged, 1 Pulz Street, Szeged, 6724 Hungary

**Keywords:** Inflammatory bowel disease, Sarcopenia, Body composition, Surgery, Postoperative complications

## Abstract

Patients with inflammatory bowel disease (IBD) are at risk of sarcopenia, which is associated with poor clinical outcomes. We conducted this study to assess whether sarcopenia predicts the need for surgery and postoperative complications in patients with IBD. We performed a systematic search of four electronic databases, last updated in March, 2019. Data from studies comparing rates of surgery and postoperative complications in sarcopenic IBD patients versus non-sarcopenic IBD patients were pooled with the random-effects models. We calculated the odds ratios (OR) with a 95% confidence interval (CI). Ten studies with a collective total of 885 IBD patients were included in our meta-analysis. Although the analysis of raw data did not reveal significant differences between the two groups with respect to the rate of surgery and postoperative complications (OR = 1.826; 95% CI 0.913–3.654; *p* = 0.089 and OR = 3.265; 95% CI 0.575–18.557; *p* = 0.182, respectively), the analysis of adjusted data identified sarcopenia as an independent predictor for both of the undesirable outcomes (OR = 2.655; 95% CI 1.121–6.336; *p* = 0.027 and OR = 6.097; 95% CI 1.756–21.175; *p* = 0.004, respectively). Thus, early detection of sarcopenia in patients with IBD is important to prevent undesirable outcomes.

**Electronic supplementary material:**

The online version of this article (10.1007/s00595-019-01893-8) contains supplementary material, which is available to authorized users.

## Introduction

The term ‘sarcopenia’ was introduced by Rosenberg in 1997 to describe an age-related decrease in skeletal muscle mass [1]. According to the recent recommendation of the European Working Group on Sarcopenia in Older People (EWGSOP), sarcopenia can be diagnosed if low lean muscle mass stands together with either low muscle strength or low physical performance [2]. In clinical practice, sarcopenia often remains unrecognized if only body mass index (BMI) is used to determine nutritional status [3]. Several studies have shown that BMI does not predict low lean muscle mass accurately and the entity of sarcopenic obesity also exists [3–5].

Sarcopenia can be assessed anatomically and functionally. Anatomically, a variety of methods and measures are used to evaluate muscle mass, including computed tomography (CT), magnetic resonance imaging (MRI), bioelectrical impedance analysis (BIA), dual-energy X-ray absorptiometry (DXA) and skeletal muscle index (SMI), appendicular SMI (ASMI), and total psoas muscle area (TPA) [2]. However, there is a considerable variance in the cut-off thresholds for sarcopenia in these diagnostic modalities, while the characteristics of the (normal) reference populations also vary [6]. Functionally, the handgrip strength test with a standard dynamometer or a physical performance test such as that using the Short Physical Performance Battery are the gold standards [2]. Sarcopenia in the elderly is often related to adverse health outcomes including a higher risk of hospitalization and mortality with associated increased healthcare costs [7, 8]. Recently, sarcopenia was implicated as a prognostic factor in a wide range of diseases, such as cancer [9, 10], chronic liver diseases [11], chronic pancreatitis [12], rheumatic diseases [13] and inflammatory bowel disease (IBD) [14].

Crohn’s disease (CD) and ulcerative colitis (UC) are the two main forms of IBD, and both are characterized by chronic inflammation of the digestive tract. Bryant et al. reported low lean muscle mass and sarcopenia in 21% and 12% of adult IBD patients, respectively [3]. In a recent systematic review, the incidence of sarcopenia was as high as 52% in CD and 37% in UC, when anatomical criteria were considered without functional strength assessment [14]. Undesired consequences of the altered body composition in IBD include bone demineralization (osteopenia and osteoporosis), inadequate response to therapy, and poor quality of life [3, 15]. Despite the associations supporting the adverse effect of sarcopenia in many diseases other than IBD, there have been only a few low-volume trials addressing this problem. Thus, we performed this meta-analysis to summarize and synthesize the results of the most up-to-date literature investigating the effect of sarcopenia, as a prognostic factor, on the need for disease-related surgery and on the characteristics of postoperative complications in patients with IBD.

## Methods

This meta-analysis was reported in accordance with the Preferred Reporting Items for Systematic Reviews and Meta-Analyses (PRISMA) Statement (Supplementary Table 1) [16]. The protocol was registered in the International Prospective Register of Systematic Reviews (PROSPERO) a priori under registration number CRD42018118517.

### Data sources and search strategy

Our search was conducted in four electronic databases using PubMed (http://www.ncbi.nlm.nih.gov/pubmed), EMBASE (https://www.embase.com), Central Cochrane Register of Controlled Trials (CENTRAL) (http://www.cochranelibrary.com) and Web of Science (www.webofknowledge.com), last updated 13 March 2019. ‘English-language’ and ‘human’ filters were applied to the search. A manual search was also done by browsing the reference lists of relevant papers and review articles to identify additional studies. The PECO items of our prognostic meta-analysis were as follows: (P) adult patients with IBD, with available body composition assessment results; (E) those who had diagnosed sarcopenia; and (C) those who did not have sarcopenia. Our outcomes (O) included the number of surgical interventions and postoperative complications. The standardized Clavien-Dindo classification tool was applied to categorize minor and major postoperative complications [17]. Major complications were defined as grade ≥ III on the Clavien-Dindo scale. Studies were identified by entering (*‘body composition’ OR sarcopenia) AND (inflammatory bowel disease or Crohn or ‘ ulcerative colitis’*) combining Medical Subject Headings (MeSH) and free-text terms.

### Study selection

After importing all references into a reference management software (EndNote X8, Clarivate Analytics, Philadelphia, PA, US), duplicates and database overlaps were removed by one of the authors (AE). To maximize the precision of selection, the remaining records were screened based on title and abstract, independently, by two of the authors (AE and PS). Finally, the same two authors verified whether the remaining full-text articles or abstracts truly fit the inclusion criteria. If there was disagreement at any stage of the selection, the opinion of a third author (PH) was sought to reach a consensus. English-language full-text articles and conference proceedings were eligible for inclusion if they met our inclusion criteria. Uncontrolled studies were excluded.

### Data extraction and quality assessment

From the included studies, two authors extracted the data according to a predefined data abstraction form: first author, year and form of publication (full-text article/abstract only), study design, sample size, and outcome (rate of patients requiring IBD-related surgery and postoperative complications). The definition of sarcopenia and the type of surgery were also recorded (Table 1). Adjusted results generated from, and covariates imputed in, multivariate logistic regression models were also collected (Supplementary Table 2).

**Table 1:** Study characteristics and measured outcomes

Author (year) study type	Number of patients (CD/UC)	Definition of sarcopenia	Type of surgery	Number of patients in the study groups (*n*)	Outcomes (number of patients)			
					Surgery	Postoperative complication	Minor postoperative complication	Major postoperative complication
Adams [23] retrosp.	90 (76/14)	Men: SMI < 52.4 cm^2^/m^2^ Women: SMI < 38.5 cm^2^/m^2^	Previous surgery, not specified	Sarcopenic (41)	21	NA	NA	NA
				Non-sarcopenic (49)	19	NA	NA	NA
Bamba [24] retrosp.	72 (43/29)	Men: SMI < 42 cm^2^/m^2^ Women: SMI < 38 cm^2^/m^2^	Intestinal resection, not specified	Sarcopenic (30)	16	NA	NA	NA
				Non-sarcopenic (42)	9	NA	NA	NA
Carvalho [25] retrosp.	58 (58/0)	Men: SMI < 52.4 cm2/m2 Women: SMI < 38.5 cm^2^/m^2^	Surgical resection, not specified	Sarcopenic (24)	7	7	NA	NA
				Non-sarcopenic (34)	17	2		
Cushing [26] retrosp.	82 (0/82)	Men: SMI < 55 cm^2^/m^2^ Women: SMI < 39 cm^2^/m^2^	Colectomy	Sarcopenic (57)	16	NA	NA	NA
				Non-sarcopenic (25)	3	NA	NA	NA
Fujikawa [27] retrosp.	69 (0/69)	Men: TPA < 567.4 mm^2^/m^2^ Women: TPA < 355.8 mm^2^/m^2^	Two- or three-stage ileal J-pouch–anal anastomosis (IPPA)	Sarcopenic (18)	NA	8	4	4
				Non-sarcopenic (51)	NA	5	3	2
O’Brien [22] retrosp	77 (52/21)^b^	In case of BMI < 25 kg/m^2^: men: SMI < 43 cm^2^/m^2^ women: SMI < 41 cm^2^/m^2^; In case of BMI ≥ 25 kg/m^2^: men: SMI < 53 cm^2^/m^2^	IPPA, ileocecectomy, hemicolectomy, colectomy, panproctocolectomy with or without ileostomy formation	Sarcopenic (30)	77	6	NA	6
				Non-sarcopenic (47)		10		10
Oh [28] retrosp.	79 (79/0)	Men: SMI < 55 cm^2^/m^2^ Women: SMI < 39 cm^2^/m^2^	Surgery, not specified	Sarcopenic (64)	11	NA	NA	NA
				Non-sarcopenic (15)	2	NA	NA	NA
Thiberge [21] retrosp.	149 (149/0)	Men: SMI < 55.4 cm^2^/m^2^ Men: SMI < 38.9 cm^2^/m^2^	Stricturoplasty, bowel resection, stoma without resection, perianal/abdominal abscess drainage, viscerolysis	Sarcopenic (50)	OR: 2.03^a^, (CI 0.98–4.26), *p *= 0.056	NA	NA	NA
				Non-sarcopenic (99)		NA	NA	NA
Zhang [29] prosp.	114 (114/0)	Men: SMI < 55 cm^2^/m^2^ Women: SMI < 39 cm^2^/m^2^	Segmental/total colectomy, ileocecal/small bowel resection	Sarcopenic (70)	NA	32	21	11
				Non-sarcopenic (44)	NA	26	25	1
Zhang [30] prosp.	99 (0/99)	Men: SMI < 55 cm^2^/m^2^ Women: SMI < 39 cm^2^/m^2^	Colectomy	Sarcopenic (27)	7	NA	NA	NA
				Non-sarcopenic (72)	7	NA	NA	NA

*CD* crohn’s disease, *UC* ulcerative colitis, *SMI* skeletal muscle index, *TPA* total psoas muscle area, *OR* odds ratio, *CI* confidence interval, *p p* value, *retrosp* retrospective, *prosp* prospective, *NA* non-available

^a^No raw data available

^b^Additional four cases of indeterminate colitis

We also collected data on patient characteristics such as age, sex, disease duration, BMI, smoking, prior use of immunomodulatory or biological therapies, and preoperatively measured laboratory parameters including hemoglobin (Hb), serum albumin level, C-reactive protein (CRP) and erythrocyte sedimentation rate (ESR) (Table 2). Two independent investigators (AE and PS) performed the quality assessment separately and any disagreements were resolved by discussion. A critical appraisal tool for prognostic studies, the Quality in Prognosis Studies (QUIPS), was used to assess the methodological quality of the studies included [18]. QUIPS covers six main domains, namely, study participation, study attrition, prognostic factor and outcome measurement, study confounding and statistical analysis, and reporting. For each item of the six domains, we used ‘yes’, ‘no’, or ‘unclear’ to assess the risk of bias. Each domain was then judged as carrying ‘low’, ‘moderate’ or ‘high’ risk of bias (Supplementary Table 3).

**Table 2:** Patient characteristics and preoperative laboratory parameters of the sarcopenic and non-sarcopenic study groups

Author (year)	Study group	Patients’ characteristics							Preoperative laboratory parameters			
		Age (years)	Male [*n* (%)]	Disease duration (years)	BMI (kg/m^2^)	Smoking [*n* (%)]	Prior use of immunomodulator [*n* (%)]	Prior use of biologics n (%)	Hemoglobin (g/dL)	Serum albumin (g/dL)	CRP (mg/L)	ESR (mm/h)
Adams [23]	Sarcopenic (41)	35 (26, 50)^a^	28 (68)	4 (2, 13)^a^	19 (18, 24)^a^	11 (27)	NA	9 (20)	NA	3.8 (3.5, 4.2)^a^	16.8 (5.1, 37.4)^a^	25 (12, 49)^a^
	Non-sarcopenic (49)	35 (26, 50)^a^	10 (20)	6 (2, 12)^a^	24 (22, 30)^a^	9 (18)	NA	6 (15)	NA	4.1 (3.8, 4.3)^a^	2.9 (1, 13.3)^a^	20 (10, 35)^a^
Carvalho [25]	Sarcopenic (24)	NA	13 (54.2)	NA	NA	NA	19 (79.2)	15 (62.5)	NA	3.49 ± 0.60^b^	NA	NA
	Non-sarcopenic (34)	NA	14 (41.2)	NA	NA	NA	26 (76.5)	19 (55.9)	NA	3.52 ± 0.60^b^	NA	NA
Cushing [26]	Sarcopenic (57)	41 ± 28^b^	43 (75.4)	2 ± 7^b^	23 ± 6^b^	2 (12.5)	20 (35.1)	17 (29.8)	11.4 ± 2.5^b^	3.4 ± 0.6^b^	50 ± 76^b^	37 ± 42^b^
	Non-sarcopenic (25)	32 ± 16^b^	10 (40)	3.5 ± 4.5^b^	26 ± 8^b^	1 (11.1)	13 (52)	9 (36)	11.3 ± 2.4^b^	3.5 ± 0.6^b^	34 ± 63.2^b^	42.5 ± 29.1^b^
Fujikawa [27]	Sarcopenic (18)	36.0 ± 17.4^b^	12 (66.6)	7.2 ± 7.9^b, g^	18.0 ± 2.9^b^	NA	NA	NA	NA	3.72 ± 0.59^b^	16 ± 25^b^	NA
	Non-sarcopenic (51)	41.2 ± 13.4^b^	33 (64.7)	8.4 ± 7.3^b, g^	21.3 ± 3.5^b^	NA	NA	NA	NA	3.87 ± 0.47^b^	7.0 ± 15^b^	NA
O’Brien [22]	Sarcopenic (30)	43 (20–80)^c^	15 (50)	NA	21 (16–37)^f^	6 (20)	20 (67)	NA	11.0 (9.8–12)^d^ 10.26 (3.7–19.6)^e^	3.35 (1.7–5.1)^c^	63.26 (0–547)^c^	NA
	Non-sarcopenic (47)	41 (21–74)^c^	31 (66)	NA	24 (19–33)^f^	7 (15)	23 (49)	NA	12.6 (10.5–13.7)^d^ 12.8 (11.4–13.8)^e^	3.53 (2.0–4.8)^c^	46.16 (0–374.7)^c^	NA
Zhang [29]	Sarcopenic (70)	28.8 ± 10.2^b^	55 (78.6)	NA	17.1 ± 2.9^b^	NA	NA	NA	10.7 ± 2.1^b^	3.6 ± 0.5^b^	29.1 ± 54.1^b^	NA
	Non-sarcopenic (44)	37.1 ± 11.6^b^	20 (45.5)	NA	19.1 ± 1.9^b^	NA	NA	NA	11.2 ± 1.7^b^	3.8 ± 0.3^b^	17.2 ± 37.4^b^	NA

*CRP* C-reactive protein, *ESR* erythrocyte sedimentation rate, *NA* non-available

^a^Median (25%, 75%)

^b^Mean ± SD

^c^Mean (range)

^d^Mean (range) for male

^e^Mean (range) for female

^f^Median (range)

^g^Measured in months

### Statistical analysis

The Comprehensive MetaAnalysis software Version 3 (Biostat, Inc., Englewood, NJ, USA) was applied to perform meta-analytical calculations with the random effects model [19]. We computed relative measures [odds ratios (ORs) and weighted mean differences (WMDs)] and event rates with 95% confidence intervals (CIs). The main outcomes, namely, surgical interventions and postoperative complications, were handled as binary variables. First, ORs from raw 2 × 2 contingency tables were pooled [19]. Peto’s OR was calculated in the case of the study of Carvalho et al. due to rare events [20]. In addition, covariate-adjusted ORs computed with multivariable logistic regression models in the individual studies were pooled [19]. For numerical variables, namely, age, disease duration, BMI, and laboratory parameters, WMDs were calculated. For differences regarding sex, smoking, prior immunomodulator and biologics use between groups, event rates were calculated.

The results of our statistical analysis are shown in tables and forest plots. All analyses were two-tailed and *p* < 0.05 was considered significant. For assessing heterogeneity, Cochrane’s *Q* and the *I*^2^ statistics were used. In the case of the *Q* statistic, *Q* exceeds the upper-tail critical value of Chi-square with the *k *− 1 degree of freedom. *I*^2^ represents the percentage of effect size heterogeneity, which cannot be explained by random chance. Based on the cochrane handbook for systematic reviews of interventions, heterogeneity was interpreted as moderate if it was between 30 and 60%, as substantial if it was between 50 and 90%, and as considerable if it was above 75% [20]. Publication bias was assessed by the visual inspection of the funnel plot, which was complemented with the Egger’s test for analysis of the need for surgical interventions [20].

## Results

### Search results

**Fig. 1:**
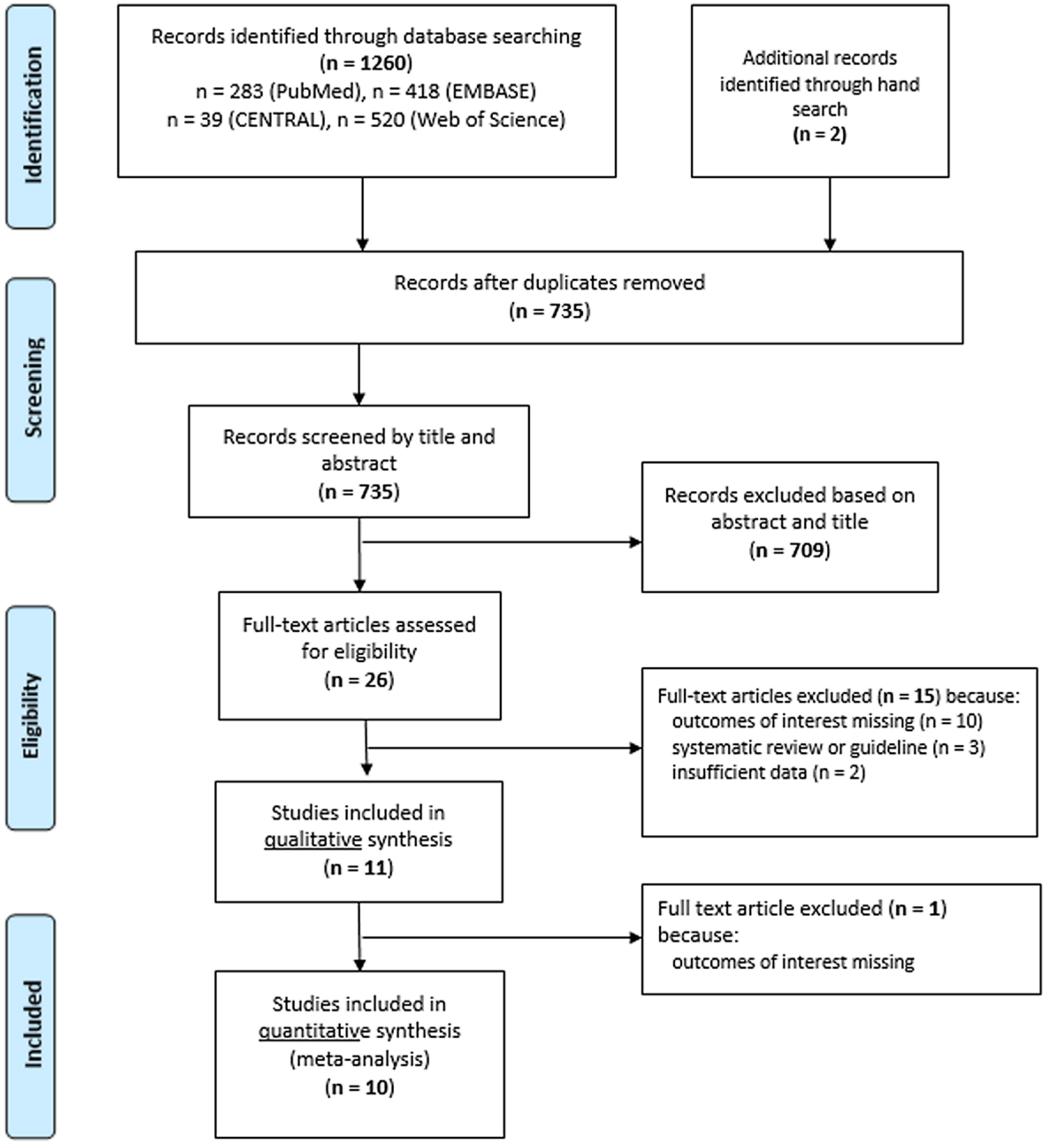
Flow diagram of the study selection process

A total of 1260 records were identified from the databases with our systematic search (PubMed: 283; EMBASE: 418; CENTRAL: 39 and Web of Science: 520 articles) (PRISMA flowchart; Fig. 1). Other two potentially eligible articles were identified by our manual search [21, 22]. After removing all duplicates, 735 records remained, 709 of which were excluded based on title and abstract screening. According to our selection criteria, 26 potentially eligible full-text articles and abstracts were considered for final inclusion, 16 of which were excluded, because they (1) failed to report the outcomes of interest (*n* = 11), (2) were systematic reviews (*n* = 3), or (3) were published only in abstract form not containing the required data (*n* = 2). Ten studies were included in the final quantitative analysis [21–30].

### Characteristics of the studies included

Table 1 summarizes the characteristics of the included studies. The trials were published between 2015 and 2018 and all studies were published in full-text form, except for one released as an abstract [28]. The majority were case–control studies and only two prospective cohort studies of Zhang et al. were eligible for meta-analysis [29, 30]. In four studies, patients with CD were recruited [21, 25, 28, 30], whereas in another three studies, only patients with UC were recruited [26, 27, 29]. In three trials, a mixed cohort of patients with CD and UC were recruited [22–24].

To quantify the degree of sarcopenia, the majority of studies measured the SMI at the level of the third lumbar vertebra on CT scan, performed within 6 months prior to surgery or in the first postoperative month. The cut-off values of the SMI defining sarcopenia ranged between 39 and 41 cm^2^/m^2^ for women and between 52 and 55 cm^2^/m^2^ for men. Bamba et al. used standardized SMI cut-off values for liver disease with a lower SMI for men (42 cm^2^/m^2^) [24]. Only one study applied TPA for sarcopenia assessment [27].

Whilst seven studies compared IBD patients with sarcopenia versus those without sarcopenia in relation to need for surgery [21, 23–26, 28, 29], only four studies published results on complications occurring in the first month after surgery [22, 25, 27, 30]. A total of 275 IBD-related operations were performed in the studies included. A high diversity of surgical interventions was registered, including stricturoplasty, bowel resection, abscess drainage, viscerolysis, ileal J-pouch–anal anastomosis, and various types of colectomy.

### Patients’ characteristics

Altogether, 885 patients with IBD were included in this meta-analysis: 571 (64.5%) with CD and 314 (35.5%) with UC. Based on the body composition measurements, 46.2% (409/885) of the patients had sarcopenia. There were significantly more men in the sarcopenic group than in the non-sarcopenic group (66.8% vs. 46.3%, *p* = 0.027; Tables 2 and 3). Patients with IBD and sarcopenia had significantly lower BMI and preoperative serum albumin levels and significantly higher CRP levels than the non-sarcopenic patients (WMD: 2.698 kg/m^2^, 95% CI 1.507–3.889, *p *< 0.001; *I*^*2*^ = 52.39%, *p* = 0.078; WMD: 3.276 g/dL, 95% CI 0.022–0.623, *p *= 0.035; *I*^*2*^ = 90.74%, *p* < 0.001 and WMD: 12.740 mg/L, 95% CI 7.154–18.326, *p *< 0.001; *I*^*2*^= 0%, *p* = 0.524, respectively). There were no significant differences in age, disease duration, smoking habits, or prior immunomodulator or biologics use between the sarcopenic and the non-sarcopenic groups (Table 3).

**Table 3:** Heterogeneity of data on the differences in patient characteristics and laboratory parameters of the sarcopenic and non-sarcopenic study groups

	Difference in means	95% CI		*p* value	Heterogeneity			
		Lower limit	Upper limit		*Q* value	*df* (*Q*)	*p* value	*I*^2^
Age	− 1.432	− 7.215	4.351	0.627	14.260	4	0.007	71.95
Disease duration (months)	− 1.441	− 5.403	2.522	0.476	0.861	2	0.650	0.0
BMI	− 2.698	− 3.889	− 1.507	**< 0.001**	8.401	4	0.078	52.39
Preoperative Hb	− 3.437	− 9.502	2.951	0.302	0.730	1	0.393	0.0
Preoperative serum albumin	− 3.276	− 0.623	− 0.022	**0.035**	53.97	5	0.000	90.74
Preoperative CRP	12.740	7.154	18.326	< **0.001**	3.204	4	0.524	0.0
Preoperative ESR	3.163	− 8.137	14.463	0.583	1.407	1	0.236	28.94

	Event rate	95% CI		*p* value	Heterogeneity			
		Lower limit	Upper limit		*Q* value	*df* (*Q*)	*p* value	*I*^2^
Sex, male: sarcopenic	0.668	0.541	0.775	0.027	11.247	5	0.047	55.44
Sex, male: non-sarcopenic	0.463	0.338	0.593		25.723	5	0.000	80.56
Smoking: sarcopenic	0.146	0.081	0.247	0.786	8.326	2	0.016	75.99
Smoking: non-sarcopenic	0.137	0.056	0.286		2.426	2	0.297	17.55
Prior immunomodulators: sarcopenic	0.593	0.411	0.752	0.400	7.544	2	0.023	73.45
Prior immunomodulators: non-sarcopenic	0.484	0.316	0.657		6.403	2	0.041	68.77
Prior biologics: sarcopenic	0.354	0.260	0.461	0.815	10.769	1	0.005	81.43
Prior biologics: non-sarcopenic	0.341	0.258	0.434		15.742	2	0.000	87.23

*BMI* body mass index, *Hg* hemoglobin, *CRP* C-reactive protein, *ESR* erythrocyte sedimentation rate, *CI* confidence interval, *df* degree of freedom

Associations significant at *p* < 0.05 vs controls shown in bold

Analyzing preoperative laboratory studies (serum albumin and CRP) and other patient characteristics (such as the rate of sarcopenia, BMI, SMI, and height) by IBD subtypes revealed that the rate of sarcopenia was significantly higher in patients with CD than in those with UC (60.7% vs. 36.7%, *p* = 0.044) [24, 29]. The preoperative serum albumin level was also significantly higher in those with CD than in those with UC (WMD: 0.337 g/dL, 95% CI 0.055–0.619, *p* = 0.019; *I*^2^ = 67.53%, *p* = 0.079) (Supplementary Table 4) [24, 29].

### Need for surgery

**Fig. 2:**
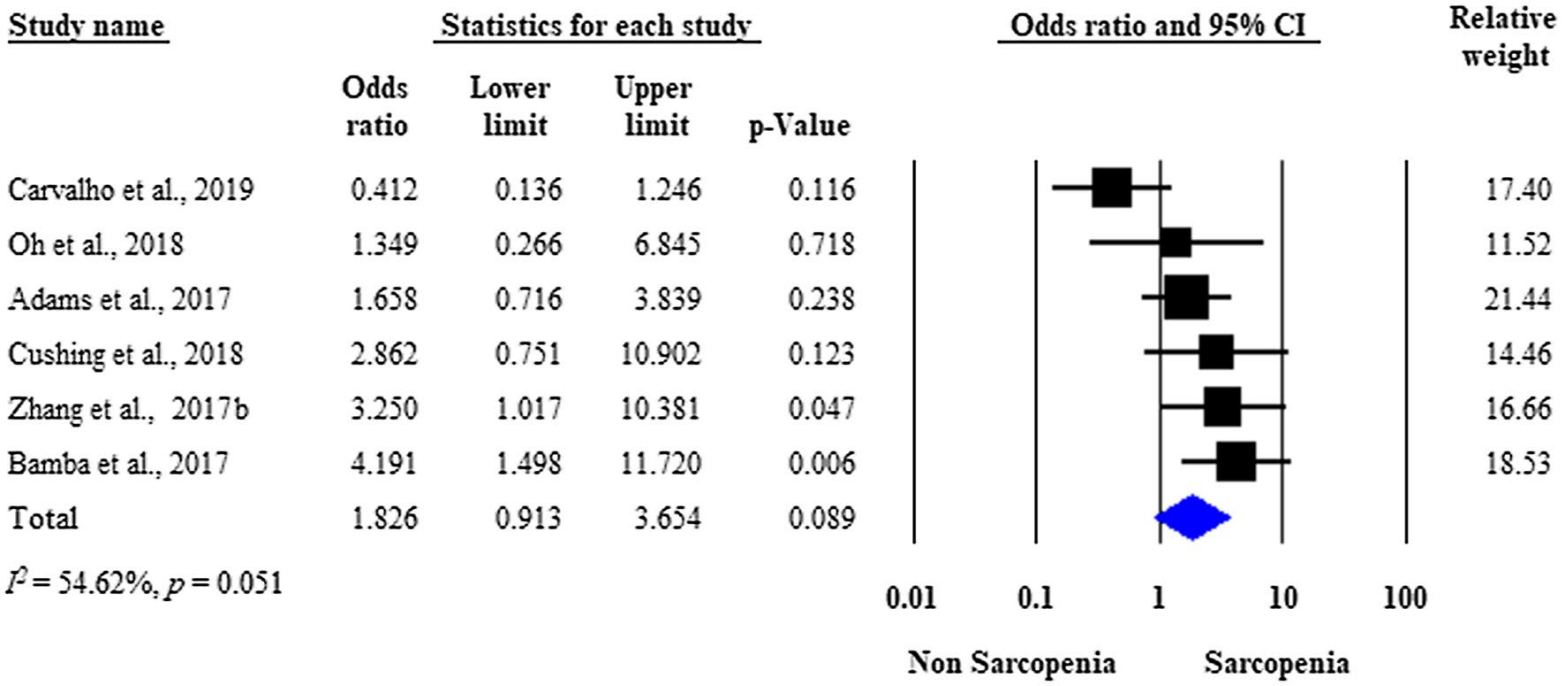
Forest plot of studies evaluating inflammatory bowel disease (IBD)-related surgical interventions in the sarcopenic and non-sarcopenic study groups (unadjusted results). Size of squares for the odds ratio reflects the weight of the trial in pooled analyses. Horizontal bars represent 95% confidence intervals

**Fig. 3:**
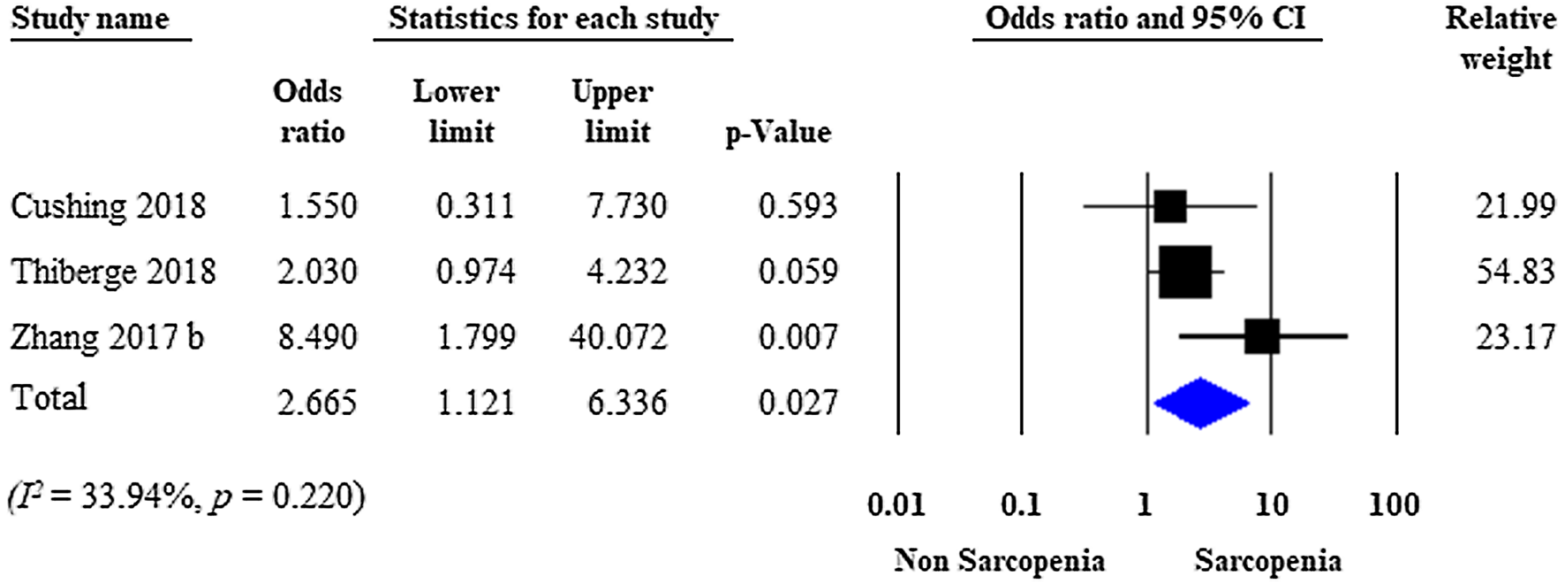
Forest plot of studies evaluating IBD-related surgical interventions in the sarcopenic and non-sarcopenic study groups (adjusted results). Size of squares for the odds ratio reflects the weight of the trial in pooled analyses. Horizontal bars represent 95% confidence intervals

Seven studies assessed the need for surgical intervention in IBD patients with versus those without sarcopenia [21, 23–26, 28, 29]. Overall, 35.5% (104/293) of the sarcopenic and 27.9% (94/336) of the non-sarcopenic patients underwent disease-related surgery, respectively. The raw data on the number of sarcopenic and non-sarcopenic patients with IBD who underwent surgery were available in six studies [23–26, 28, 29]. Analysis of the unadjusted data showed no significant difference between the two groups with respect to the need for surgical interventions (unadjusted OR: 1.826; 95% CI 0.913–3.654; *p* = 0.089). Moderate heterogeneity was detected across the studies (*I*^*2*^ = 54.62%, *p* = 0.051) (Fig. 2). Three studies were adjusted for significant covariates [21, 26, 29]. After pooling the adjusted ORs together, sarcopenia proved to be an independent predictor of the need for surgery (adjusted OR: 2.665; 95% CI 1.121–6.336; *p* = 0.027), with moderate between-study heterogeneity (*I*^*2*^ = 33.94%, *p* = 0.220) (Fig. 3).

**Fig. 4:**
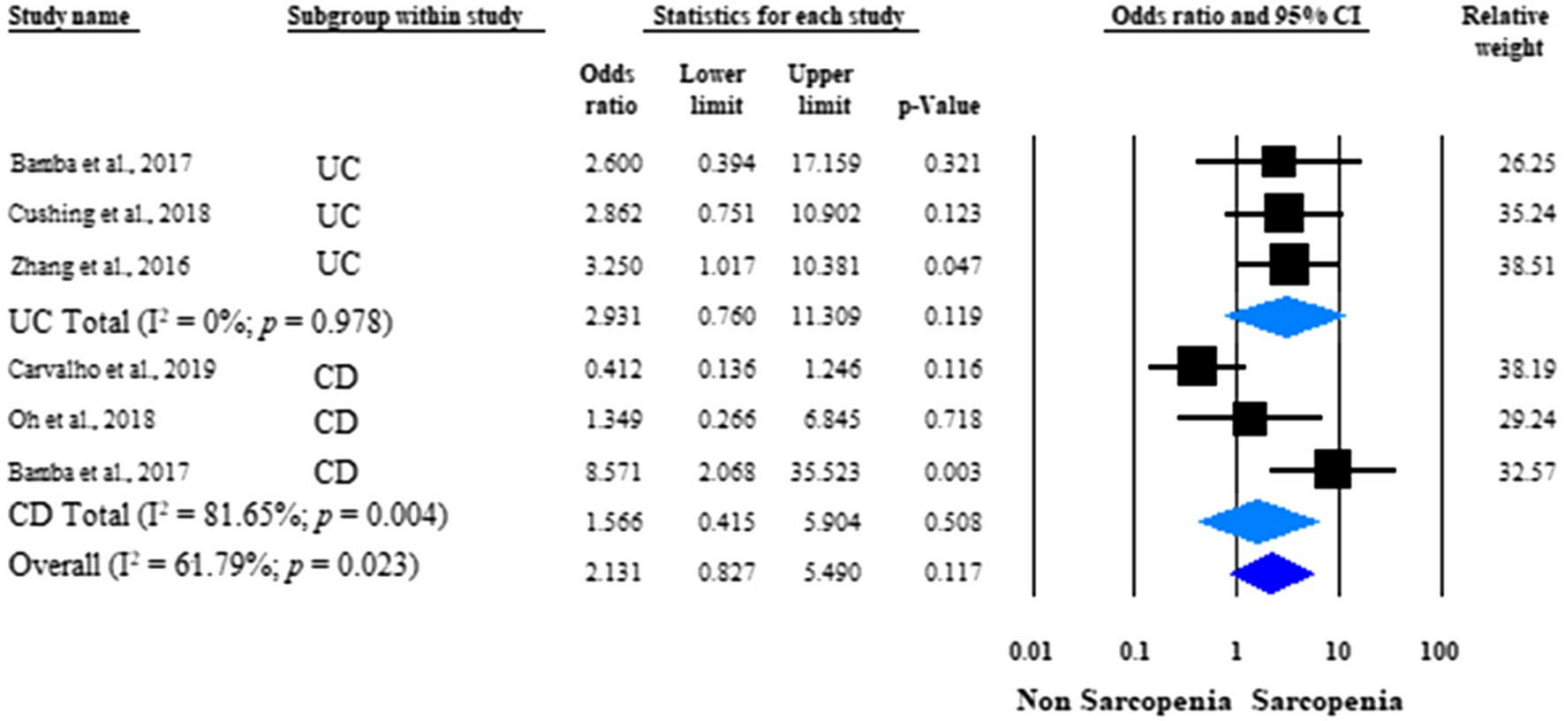
Forest plots of studies evaluating the effect of sarcopenia on disease-related surgical interventions in the Crohn’s disease (CD) and ulcerative colitis (UC) patient populations (unadjusted results). Size of squares for odds ratio reflects weight of trial in pooled analysis. Horizontal bars represent 95% confidence intervals. *UC* ulcerative colitis, *CD* Crohn’s disease

When the data of the CD and UC patients were analyzed separately, no significant difference was found between the sarcopenic and non-sarcopenic groups in the rate of surgical intervention (unadjusted OR: 1.566; 95% CI 0.415–5.904; *p* = 0.508; *I*^*2*^ = 81.65%, *p* = 0.004 and unadjusted OR: 2.931; 95% CI 0.760–11.309; *p* = 0.119; *I*^*2*^ = 0.0%, *p* = 0.978, respectively) (Fig. 4).

### Postoperative complications

**Fig. 5:**
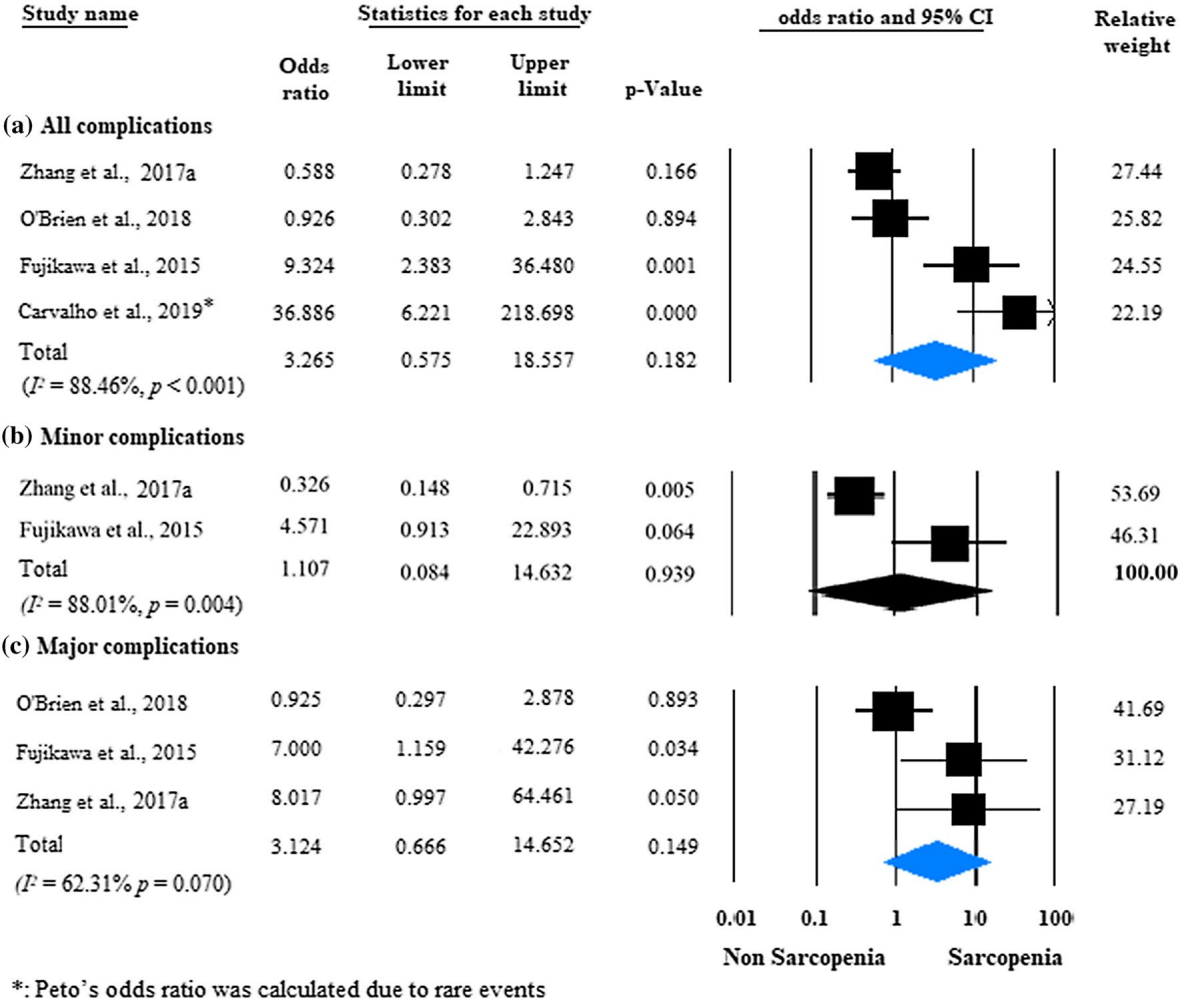
Forest plot of studies evaluating postoperative complications in the sarcopenic and non-sarcopenic patients with IBD (unadjusted results). **a** All complications; **b** minor complications were defined as grade I–II; **c** major complications were defined as grade ≥ III on the Clavien-Dindo scale. Size of squares for the odds ratio reflects the weight of the trial in pooled analyses. Horizontal bars represent 95% confidence intervals

**Fig. 6:**
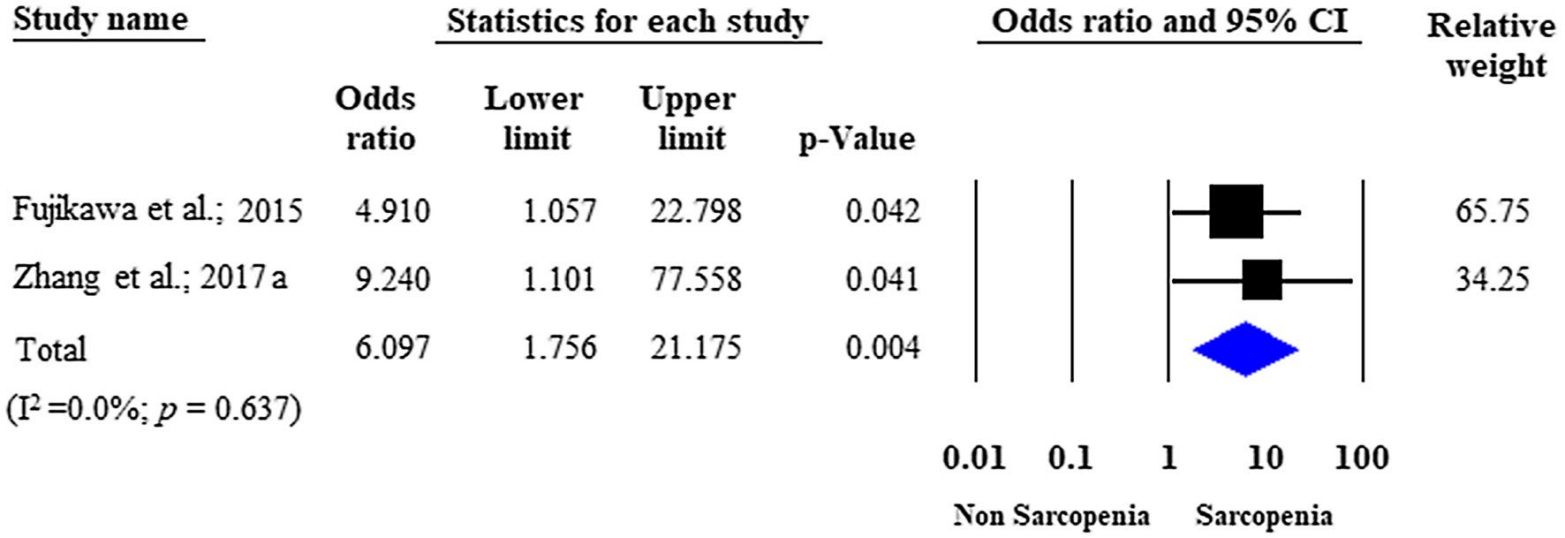
Forest plot of studies evaluating postoperative complications in the sarcopenic and non-sarcopenic patients with IBD considering all complications (adjusted results). Size of squares for the odds ratio reflects the weight of the trial in pooled analyses. Horizontal bars represent 95% confidence intervals

Four studies were eligible for analysis of postoperative complications in IBD patients with versus those without sarcopenia [22, 25, 27, 30] (Fig. 5a). Postoperative complications developed in 37.3% (53/142) of the sarcopenic patients versus 24.4% (43/176) of the non-sarcopenic group, without a significant difference between the two groups (unadjusted OR: 3.265; 95% CI 0.575–18.557; *p *= 0.182; *I*^*2*^ = 88.46%, *p* < 0.001). Two studies were adjusted for significant covariates [27, 30]. After pooling the adjusted ORs together, sarcopenia proved to be an independent predictor of postoperative complications (adjusted OR = 6.097; 95% CI 1.756–21.175; *p* = 0.004), with negligible between-study heterogeneity (*I*^*2*^ = 0.0%, *p* = 0.637) (Fig. 6).

Performing subgroup analysis based on the Clavien-Dindo grading system, sarcopenia was not a risk factor of minor or major postoperative complications (unadjusted OR: 1.107; 95% CI: 0.084-14.632; *p* = 0.939, and unadjusted OR: 3.124; 95% CI: 0.666-14.652; *p* = 0.149, respectively) (Fig. 5b, c). Considerable heterogeneity was observed in the analyses of overall, minor, and major postoperative complications (*I*^*2*^ = 88.46%, *p* < 0.001; *I*^*2*^ = 88.01%, *p* = 0.004; and *I*^*2*^ = 62.31%, *p* = 0.070, respectively).

### Risk of bias assessment

Supplementary Figs. 1 and 2 summarize the results of the risk of bias assessment of the individual studies and Supplementary Fig. 3 shows the adapted QUIPS tool. The main domain ‘study attrition’ and further selected items not suitable for the studies included in the meta-analysis were omitted. Based on our analysis, the study of Thiberge et al. obtained the best scores with only one moderate risk of bias, while the study of Oh showed the worst results with two domains classified as carrying high and one domain classified as carrying a moderate risk of bias. All the studies included were assessed as carrying a high risk of bias in at least one domain. The domain of ‘prognostic factor measurement’ was the best rated, as all the included studies were judged to carry a low risk of bias. In contrast, 90% of the studies showed a high risk of bias in the term of ‘study confounding’, since they did not report how the important confounders were adjusted for and whether an appropriate method was used for handling missing data. In all the studies evaluated, ‘study participation’ and ‘outcome measurement’ domains carried low or moderate risk of bias. Most of the studies (90%) detailed statistical analysis properly and were, therefore, awarded a green notation in this regard.

### Publication bias

Based on visual assessment of the funnel plot (symmetric) and the Egger’s test result (*p* = 0.91), a ‘small-study effect’ is unlikely to occur in the analysis of the need for surgical intervention (Supplementary Fig. 3).

## Discussion

Sarcopenia is relevant for patients with IBD as it can lead to poor outcomes, such as bone demineralization with consequential pathological fractures, hospitalization, reduced mobility, and compromised quality of life [31]. Apart from the increased level of pro-inflammatory mediators, inadequate calorie intake, malabsorption, and protein-losing enteropathy, different pharmacological and surgical treatments may also impair nutritional status in IBD [15]. However, few studies have evaluated the effect of sarcopenia as a prognostic factor of surgical outcomes.

Our meta-analysis found only seven studies with controversial results on surgical interventions for sarcopenic patients with IBD. Although we did not detect a significant difference between the rate of surgical interventions for the sarcopenic patients versus the non-sarcopenic patients with IBD when unadjusted data were pooled, our analysis of covariate-adjusted data identified sarcopenia as an independent predictor of surgical interventions in patients with IBD. A potential explanation for this phenomenon could be the effect of bias (especially selection bias), which masked the true difference in the unadjusted analysis (Figs. 2 and 5), but was reduced, at least partly, with the introduction of multivariate models (Figs. 3 and 6). Regarding IBD subtype (CD or UC), no significant difference was observed in surgical interventions, although there was a trend towards a more frequent need for surgery in sarcopenic patients with UC.

Similarly, we could only detect significant differences between the groups with respect to postoperative complications when adjusted data were analyzed. This finding is consistent with previous meta-analyses on patients who underwent oncological abdominal surgery, where radiologically proven sarcopenia was associated with a significant increase in major postoperative complications as well as in 30-day mortality [32, 33]. In our meta-analysis, most of the sarcopenic patients were men. Interestingly, a previous study identified sarcopenia as a predictor of a subsequent surgery only in women with IBD [34]. Other clinical features such as age, disease duration, smoking, and prior therapies did not differ considerably between the groups. We also found lower BMI and serum albumin levels and higher CRP levels in the sarcopenic group. It should be noted that measuring these parameters can be helpful in the prediction of sarcopenia. Since cross-sectional imaging is frequently ordered preoperatively, the results of CT or MRI scans would provide a more accurate estimation of lean muscle mass.

Although the consequences of malnutrition, such as poor bone health, delayed puberty and growth failure, are more prevalent in pediatric patients with IBD, our meta-analysis highlighted that the assessment of body composition is also necessary in adult patients with IBD [35]. The importance of evaluating the nutritional status of an IBD patient was emphasized in the recent European Crohn’s and Colitis Organisation (ECCO) guidelines [36, 37]. Malnutrition and hypalbuminaemia are listed as the main risk factors of postoperative complications, including anastomotic leak, peritonitis, and intra-abdominal septic complications [36]. Prior to surgery, not only the responsible adjustment of medical therapy, such as weaning off steroid treatment if possible, but also preoperative enteral or parenteral nutritional support may help to reduce the risk of surgical and postoperative complications [38–42].

Several limitations of this meta-analysis must be considered. First, the vast majority of included studies were retrospective with a small sample size and a wide variety of surgical interventions, raising concerns about imprecision and indirectness. Second, none of the included studies used EWGSOP criteria to assess functional loss of muscle strength measurement. Third, only one study assessed the effect of nutritional therapy, in which preoperative enteral nutrition was a protective factor against major postoperative complications [30]. Furthermore, minor differences were observed with respect to the covariates imputed in the logistic regression models, which may affect our results (Supplementary Table 2). As heterogeneity tests indicated homogeneous datasets (Figs. 3 and 6), we do not suspect rough distortion. Finally, there was considerable heterogeneity in cut-off points for the sarcopenia definition regarding ethnicity, carrying the potential of under- or overestimating the sarcopenia rate in the included studies.

The main strength of our work is its novelty, but we must mention the foremost systematic review by Ryan et al. [14] investigating the prognostic role of sarcopenia in the outcomes of surgery for IBD. Our review revealed the need for longitudinal observational studies in this field. The highly transparent and reproducible methodology of this work was ensured by strictly adhering to the rules and recommendations of the PRISMA guidelines.

In conclusion, the findings of our analysis have implications for practice, particularly in the promotion of preoperative individualized risk prediction. In addition to simple anthropometric tests, anatomical and functional measurements should be performed. The SMI, measured on a CT scan, can be used as an objective assessment tool to identify sarcopenia in patients with IBD. To interpret the body composition and nutritional status of patients with IBD, a multidisciplinary approach is recommended. Education on nutritional issues is best provided by well-trained dietitians with a special interest in IBD. Since sarcopenia may be reversible with adequate nutritional support, targeted preoperative risk reduction strategies are recommended to optimize surgical outcomes. Further research through large prospective cohort studies is needed to confirm our findings and conclusions.

## Electronic supplementary material

Below is the link to the electronic supplementary material.

Supplementary Table 1 PRISMA checklist for preferred reporting items for systematic reviews and meta-analyses (DOC 64 kb)

Supplementary Table 2 (DOCX 14 kb)

Supplementary Table 3 (DOCX 26 kb)

Supplementary Table 4 (DOCX 23 kb)

Supplementary Fig. 1 Risk of bias summary for studies on sarcopenia as a prognostic factor for surgery and postoperative complications (QUIPS tool) green notation: low risk of bias; yellow notation: moderate risk of bias; red notation: high risk of bias (PNG 181 kb)

Supplementary Fig. 2 Risk of bias graph for studies on sarcopenia as a prognostic factor for surgery and postoperative complications (QUIPS tool) (PNG 23 kb)

Supplementary Fig. 3 Funnel plot of studies comparing the need for surgical intervention in sarcopenic versus non-sarcopenic IBD patients with pseudo 95% confidence limits. Each circle indicates one study with its standard error indicating the weight of the study and its odds ratio. The dotted lines represent 95% confidence interval to visualize the symmetry around the pooled estimate (PNG 11 kb)

## Abbreviations

ASMI: Appendicular skeletal muscle index
BMI: Body mass index
CD: Crohn’s disease
CI: Confidence interval
CRP: C-reactive protein
CT: Computed tomography
ECCO: European Crohn’s and colitis organisation
ESR: Erythrocyte sedimentation rate
EWGSOP: European Working Group on sarcopenia in older people
Hb: Hemoglobin
IBD: Inflammatory bowel disease
OR: Odds ratio
SMI: Skeletal muscle index
TPA: Total psoas muscle area
UC: Ulcerative colitis
WMD: Weighted mean difference
