# Supplementary material for: Sarcopenia as an independent predictor of the surgical outcomes of patients with inflammatory bowel disease: a meta-analysis
Source: Surg Today. 2019 Oct 15;50(10):1138–50. doi: 10.1007/s00595-019-01893-8 (PMC7501129; doi:10.1007/s00595-019-01893-8)
Supplement: Supplementary file 2 — Supplementary Table 2 (DOCX 14 kb) [file 595_2019_1893_MOESM2_ESM.docx]

**Supplementary Table 2** Confounding factors entered in multivariate logistic regression models in each study

| **Author (year)** | **Adjusted variates** |
| --- | --- |
| Adams et al. (2017) | previous surgery, sarcopenia, serum albumin, CRP |
| Bamba et al. (2017) | CRP, serum albumin, sarcopenia, disease type (CD/UC) |
| Carvalho et al. (2019) | sarcopenia, serum albumin, corticosteroid use, SMI |
| Cushing et al. (2018) | age, gender, serum albumin, pancolitis, CRP, BMI |
| Fujikawa et al. (2015) | total protein, operative blood loss, sarcopenia, PNI |
| O’Brien et al. (2018) | sarcopenia, myosteatosis, age (> 65 years), CCI ≥ 3, NLR category > 5 |
| Oh et al. (2018) | NA |
| Thiberge et al. (2018) | sarcopenia, gender, disease behaviour, biological therapy |
| Zhang et al. (2017a) | sarcopenia, decreased SMI, preoperative enteral nutrition, serum albumin (< 35 g/L), stoma creation |
| Zhang et al. (2017b) | SMA, SMI, sarcopenia, BMI, MFI |

CD: Crohn’s disease; UC: Ulcerative Colitis; SMI: skeletal muscle index; TPA: total psoas muscle area; NA: non-available; CRP:C-reactive protein; BMI: body mass index; PNI: prognostic nutritional index; CCI: Charlson Comorbidity index; NLR: neutrophil-lymphocyte ratio, SMA: skeletal muscle area; MFI: mesenterial fat index
