# Supplementary material for: Sarcopenia as an independent predictor of the surgical outcomes of patients with inflammatory bowel disease: a meta-analysis
Source: Surg Today. 2019 Oct 15;50(10):1138–50. doi: 10.1007/s00595-019-01893-8 (PMC7501129; doi:10.1007/s00595-019-01893-8)
Supplement: Supplementary file 3 — Supplementary Table 3 (DOCX 26 kb) [file 595_2019_1893_MOESM3_ESM.docx]

**Supplementary Table 3** Quality in prognosis studies (QUIPS) tool (adapted for the study)

| **Modified QUIPS items** | **Adams 2017** | **Bamba 2017** | **Carvalho 2019** | **Cushing 2018** | **Fujikawa 2016** | **Oh 2018** | **O’Brien**  **2018** | **Thiberge 2018** | **Zhang 2017a** | **Zhang 2017b** |
| --- | --- | --- | --- | --- | --- | --- | --- | --- | --- | --- |
| **1. Study Participation** | | | | | | | | | | |
| Source of target population adequately described for key characteristics including type of the IBD, age, sex, cause of hospitalization | yes | yes | yes | yes | yes | yes | yes | yes | yes | yes |
| The method used to identify the study population is described adequately | yes | yes | yes | yes | yes | yes | yes | yes | yes | yes |
| Recruitment period is stated | yes | yes | yes | no | yes | no | yes | yes | yes | yes |
| Place of recruitment (setting and location) is stated | yes | yes | yes | yes | yes | yes | yes | yes | yes | yes |
| Inclusion and exclusion criteria are clearly defined | yes | yes | yes | yes | yes | yes | yes | yes | yes | yes |
| There is adequate participation in the study by eligible individuals >80% a1 | NA | NA | NA | NA | NA | NA | NA | NA | NA | NA |
| Baseline characteristics of the participants were collected including BMI, disease type (CD/UC) and duration, disease location or phenotype, previous medications | yes | yes | yes | yes | yes | yes | yes | yes | yes | yes |
| **Summary: Study participation** | **Low** | **Low** | **Low** | **Moderate** | **Low** | **Moderate** | **Low** | **Low** | **Low** | **Low** |
| **2. Study Attrition** | | | | | | | | | | |
| All the included patients have outcome data, or the maximum dropout rate was 20% a1 | NA | NA | NA | NA | NA | NA | NA | NA | NA | NA |
| Attempts to collect information on participants who dropped out a1 | NA | NA | NA | NA | NA | NA | NA | NA | NA | NA |
| Reasons for lost to follow-up are given a1 | NA | NA | NA | NA | NA | NA | NA | NA | NA | NA |
| Outcome and prognostic factor information for those lost to follow-up a1 | NA | NA | NA | NA | NA | NA | NA | NA | NA | NA |
| There are no important differences between participants who completed the study and those who did not a1 | NA | NA | NA | NA | NA | NA | NA | NA | NA | NA |
| **Summary: Study Attrition** |  |  |  |  |  |  |  |  |  |  |

| **3. Prognostic Factor Measurement** | | | | | | | | | | |
| --- | --- | --- | --- | --- | --- | --- | --- | --- | --- | --- |
| Definition of sarcopenia and the method of measurement are clearly described | yes | yes | yes | yes | yes | yes | yes | yes | yes | yes |
| Method of measuring of sarcopenia is valid and reliable | yes | yes | yes | yes | yes | yes | yes | yes | yes | yes |
| Continuous variables (SMI, SMM, ASMI, TPA) are reported or appropriate cut points are used | yes | yes | yes | yes | yes | yes | yes | yes | yes | yes |
| Method and setting of measuring sarcopenia are the same for all study participants | yes | yes | yes | yes | yes | yes | yes | yes | yes | yes |
| All the included participants have complete data for the sarcopenia | yes | yes | yes | yes | yes | yes | yes | yes | yes | yes |
| Appropriate method was used to replace the missing data on sarcopenia a2 | NA | NA | NA | NA | NA | NA | NA | NA | NA | NA |
| **Summary: Sarcopenia Measurement** | **Low** | **Low** | **Low** | **Low** | **Low** | **Low** | **Low** | **Low** | **Low** | **Low** |
| **4. Outcome Measurement** | | | | | | | | | | |
| Outcome: need for surgery defined as any kind of disease related abdominal surgery | yes | yes | yes | yes | NA | yes | yes | yes | NA | yes |
| Outcome: postoperative complication defined according to the Clavien-Dindo classification | NA | NA | no | NA | no | NA | yes | NA | yes | NA |
| Valid and Reliable Measurement of Outcome b1 | NA | NA | NA | NA | NA | NA | NA | NA | NA | NA |
| Method and Setting of Outcome Measurement are the same for all study participants | yes | yes | yes | yes | yes | yes | yes | yes | yes | yes |
| **Summary: Outcome Measurement** | **Low** | **Low** | **Moderate** | **Low** | **Moderate** | **Low** | **Low** | **Low** | **Low** | **Low** |
| **5. Study Confounding** | | | | | | | | | | |
| *Age*, as an important confounder is measured: | yes | yes | yes | yes | yes | yes | yes | yes | yes | no |
| *BMI*, as an important confounder is measured: | yes | yes | no | yes | yes | yes | yes | yes | yes | yes |
| *Serum albumin level*, as an important confounder is measured: | yes | yes | yes | yes | yes | no | yes | yes | yes | yes |

| The measured confounding factors are clearly defined | no | yes | yes | yes | yes | no | yes | yes | yes | yes |
| --- | --- | --- | --- | --- | --- | --- | --- | --- | --- | --- |
| Valid and Reliable Measurement of Confounders | yes | yes | yes | yes | yes | yes | yes | yes | yes | yes |
| Method and Setting of Confounding Measurement are the same for all study participants | yes | yes | yes | yes | yes | yes | yes | yes | yes | yes |
| An appropriate method was used for replacing missing confounder data (e.g. imputation) | unclear | unclear | unclear | unclear | unclear | unclear | unclear | yes | unclear | unclear |
| Important confounders are accounted for in the study design (e.g. initial assembly of comparable groups or matching) | no | no | no | no | no | no | no | no | no | no |
| Confounders considered important by the authors of the article, are accounted for in the analysis (e.g. appropriate adjustment, stratification, multivariate regression) | yes | yes | yes | yes | yes | no | yes | yes | yes | yes |
| **Summary: Study Confounding** | **High** | **High** | **High** | **High** | **High** | **High** | **High** | **Moderate** | **High** | **High** |
| **6. Statistical Analysis and Reporting** | | | | | | | | | | |
| The analytical strategy is described in detail, the adequacy of the analysis can be judged | yes | yes | yes | yes | yes | no | yes | yes | yes | yes |
| Strategy for model building is appropriate and is based on a conceptual framework or model b2 | NA | NA | NA | NA | NA | NA | NA | NA | NA | NA |
| The results are reported in detail, no selective reporting can be observed | yes | yes | yes | yes | yes | no | yes | yes | yes | yes |
| **Summary: Statistical Analysis and Presentation** | **Low** | **Low** | **Low** | **Low** | **Low** | **High** | **Low** | **Low** | **Low** | **Low** |

Items are rated as yes (low risk of bias), unclear (unknown risk of bias) or no (high risk of bias). If one of the items was rated as unclear or no, then the specific main item was considered to carrying moderate risk of bias. If two or more of the items were rated as unclear or no, then the specific main item was considered to carrying high risk of bias.

NA: not applicable due to study design; a1: items only applicable in prospective studies. As all the included studies were retrospective or the study population was retrospectively identified from a prospectively maintained database. Therefore, these items were not assessed.; a2: Only those patients for whom data on sarcopenia were available were included in the studies. Therefore, this item was not assessed.; b1: not applicable item, since we examined only well defined, hard endpoints.; b2: not applicable item, since none of the included studies aimed to build a model.
