# Supplementary material for: Sarcopenia as an independent predictor of the surgical outcomes of patients with inflammatory bowel disease: a meta-analysis
Source: Surg Today. 2019 Oct 15;50(10):1138–50. doi: 10.1007/s00595-019-01893-8 (PMC7501129; doi:10.1007/s00595-019-01893-8)
Supplement: Supplementary file 4 — Supplementary Table 4 (DOCX 23 kb) [file 595_2019_1893_MOESM4_ESM.docx]

|  | **Difference in means** | **95% CI** | | ***p* value** | **Heterogeneity** | | | |
| --- | --- | --- | --- | --- | --- | --- | --- | --- |
|  |  | **Lower limit** | **Upper limit** |  | **Q value** | **df (Q)** | ***p*-value** | **I^2^** |
| **Preoperative serum albumin** | 0.337 | 0.055 | 0.619 | **0.019** | 3.080 | 1 | 0.079 | 67.53 |
| **Preoperative CRP** | -0.506 | -1.688 | 0.676 | 0.402 | 0.007 | 1 | 0.933 | 0.0 |
| **BMI** | -0.251 | -2.304 | 1.802 | 0.811 | 6.574 | 1 | 0.010 | 84.79 |
| **Height** | 3.159 | -3.396 | 9.714 | 0.345 | 9.372 | 1 | 0.002 | 89.33 |
| **SMI** | -2.199 | -15.830 | 11.431 | 0.752 | 26.629 | 1 | 0.000 | 96.25 |
|  | **Event rate** | **95% CI** | | ***p* value** | **Heterogeneity** | | | |
|  |  | **Lower limit** | **Upper limit** |  | **Q value** | **df (Q)** | ***p*-value** | **I^2^** |
| **CD patients with sarcopenia** | 0.607 | 0.444 | 0.749 | **0.044** | 0.178 | 1 | 0.673 | 0.0 |
| **UC patients with sarcopenia** | 0.367 | 0.225 | 0.536 |  | 5.831 | 1 | 0.016 | 85.85 |

BMI: body mass index; CRP: C-reactive protein; SMI: skeletal muscle index; CI: confidence interval; df: degree of freedom; associations significant at p < 0.05 vs controls shown in bold
