# Supplementary figures and images for: Sarcopenia as an independent predictor of the surgical outcomes of patients with inflammatory bowel disease: a meta-analysis
Source: Surg Today. 2019 Oct 15;50(10):1138–50. doi: 10.1007/s00595-019-01893-8 (PMC7501129; doi:10.1007/s00595-019-01893-8)

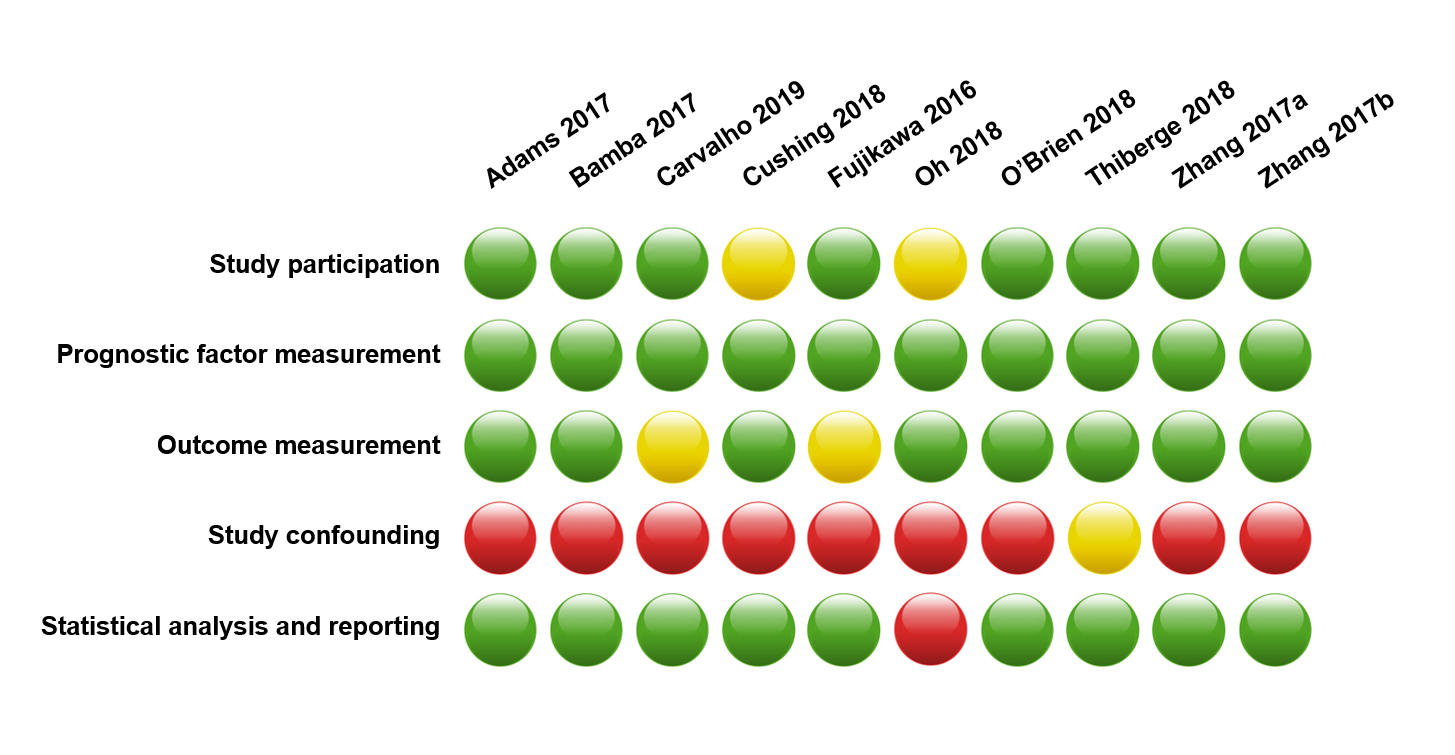

Supplement: Supplementary file 5 — Supplementary Fig. 1 Risk of bias summary for studies on sarcopenia as a prognostic factor for surgery and postoperative complications (QUIPS tool) green notation: low risk of bias; yellow notation: moderate risk of bias; red notation: high risk of bias (PNG 181 kb) [file 595_2019_1893_MOESM5_ESM.png]

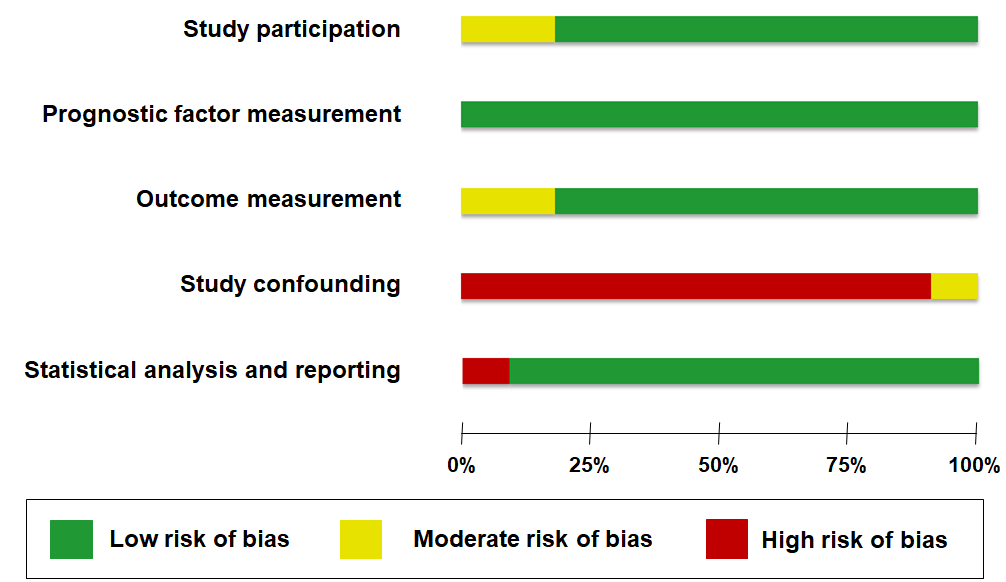

Supplement: Supplementary file 6 — Supplementary Fig. 2 Risk of bias graph for studies on sarcopenia as a prognostic factor for surgery and postoperative complications (QUIPS tool) (PNG 23 kb) [file 595_2019_1893_MOESM6_ESM.png]

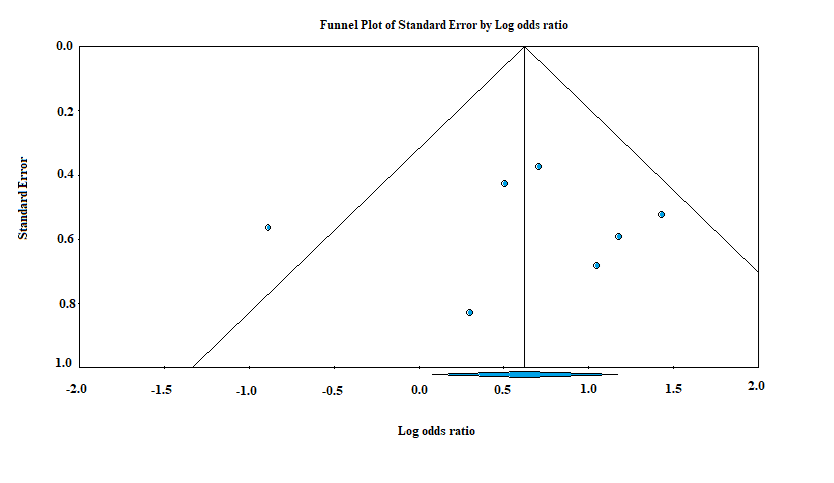

Supplement: Supplementary file 7 — Supplementary Fig. 3 Funnel plot of studies comparing the need for surgical intervention in sarcopenic versus non-sarcopenic IBD patients with pseudo 95% confidence limits. Each circle indicates one study with its standard error indicating the weight of the study and its odds ratio. The dotted lines represent 95% confidence interval to visualize the symmetry around the pooled estimate (PNG 11 kb) [file 595_2019_1893_MOESM7_ESM.png]
